# A YSK-Type Dehydrin from *Nicotiana tabacum* Enhanced Copper Tolerance in *Escherichia coli*

**DOI:** 10.3390/ijms232315162

**Published:** 2022-12-02

**Authors:** Jinran Dai, Lirou Shen, Jin Zhou, Xinyu Liu, Suiyun Chen

**Affiliations:** 1 School of Ecology and Environmental Science, Yunnan University, Kunming 650504, China; 2 Biocontrol Engineering Research Center of Plant Disease and Pest, Biocontrol Engineering Research Center of Crop Disease and Pest, Kunming 650504, China; 3 School of Life Sciences, Yunnan University, Kunming 650504, China

**Keywords:** dehydrin, NtDHN17, copper toxicity, *E. coli*, protein aggregation

## Abstract

Copper is an essential micronutrient for the maintenance of normal cell function but is toxic in excess. Dehydrins are group two late embryogenesis abundant proteins, which facilitate plant survival in harsh environmental conditions. Here, a YSK-type dehydrin, *NtDhn17*, was cloned from *Nicotiana tabacum* under copper toxicity and characterized using a heterologous expression system and in vitro or in vivo experiments and exhibited characteristics of intrinsic disorder during in vitro analyses. Heterologous expression of NtDHN17 enhanced the tolerance of *E. coli* to various metals, osmotic, and oxidative stress. NtDHN17 showed no Cu^2+^-binding properties in vivo or in vitro, indicating that metal ion binding is not universal among dehydrins. In vitro and in vivo experiments suggested that NtDHN17 behaved as a potent anti-aggregation agent providing strong protection to aggregated proteins induced by excess copper ions, an effect dependent on the K-segment but not on the Y- or S-segments. In summary, the protective role of NtDHN17 towards *E. coli* under conditions of copper toxicity may be related to anti-aggregation ability rather than its acting as an ion scavenger, which might be a valuable target for the genetic improvement of resistance to heavy metal stresses in plants.

## 1. Introduction

Low concentrations of copper (Cu) are essential for growth and development in many organisms, from bacteria and plants to humans [1,2]. However, pollution from heavy metals, such as copper, zinc, lead and cadmium, has increased over the past decade due to increasing population, industrialization and agricultural usage [3]. Excessive copper can be toxic, causing oxidative damage to macromolecules. Copper ions promote the production of reactive oxygen species (ROS), such as the superoxide anion and the hydroxyl radical, which modify structures and/or functions of biomolecules [4]. Dehydrins are group two late embryogenesis abundant (LEA) proteins which participate in the prevention of protein or membrane aggregation, bind metal ions and maintain enzyme activity under various environmental stresses. Numerous studies have investigated the metal-ion-binding and ROS suppressing properties of dehydrins under environmental stresses [5,6]. The SK-type dehydrins showed remarkable capability for metal binding and reducing the production of ROS. The citrus-derived SK-type dehydrin *CuCOR15* binds multiple metal ions [7], and AtHIRD11 binds metal and inhibits the resulting ROS generation by excess metal ions [8].

The balance between the re-folding and degradation of misfolded proteins is critical for cell viability [9] and copper has been shown to promote protein aggregation, leading to proteotoxic stress and cell death [10,11]. Copper toxicity has been linked to the inactivation of enzyme and cellular pathways [12,13], and dehydrins showed a protective role in maintaining enzyme activity. AtHIRD11 restored the activity of lactate dehydrogenase (LDH) inactivated by Cu^2+^ [14], and wheat DHN-5 maintained LDH and β-glucosidase activities under various stresses in vitro [15].

Little is known about the copper toxicity protective function of dehydrin apart from the metal-ion-binding and ROS scavenger properties. A cDNA clone of dehydrin, *NtDhn17*, was isolated from *Nicotiana tabacum* (L.) ‘K326′ and treated with dry mycelium of *Penicillium chrysogenum* (DMP) during the current study. Protective effects of *NtDhn17* were tested in *E. coli* cells. Over-expression of *NtDhn17* improved the growth of *E.coli* cells under copper toxicity, not through copper efflux system. IMAC (Immobilized metal ion affinity chromatography) analysis revealed no binding of NtDHN17 to Cu^2+^ either in vivo or in vitro, in contrast to the SK type dehydrins. Cells possess an extensive network of defense mechanisms to maintain proteome integrity and protein homeostasis (proteostasis) [16,17], and the contribution of NtDHN17 and its conserved segments was tested in vitro using insoluble proteins from *E. coli* in which aggregation was induced by copper.

Dehydrins are vital to the response mechanisms of plants to environmental stresses [18], and individual dehydrins respond to different stimuli [6]. The current study gives insights into NtDHN17 mechanisms in response to copper toxicity and increases our understanding of biochemical, physiological and biological roles in stress management.

## 2. Results

### 2.1. Isolation and Sequence Analysis of the NtDhn17 Gene and NtDHN17 Protein

Primers were designed to clone the full ORF of the *NtDhn17* gene from a cDNA library constructed from mRNAs in leaves after treatment with 7.5% DMP [19] based on previously reported sequence information of the *NtERD10B* gene and partial CDS of the *NtERD10B* gene (AB049336.1) [20]. The gene of interest is referred to as *NtDhn17* to emphasize its novel function relative to NtERD10B. *Dhn* indicates that this gene belongs to the dehydrin family, and 17 indicates the protein’s molecular weight. The deduced protein consisted of 169 amino acids with a molecular weight of 17,773.1 Da and a theoretical pI of 6.81.

**Figure 1:**
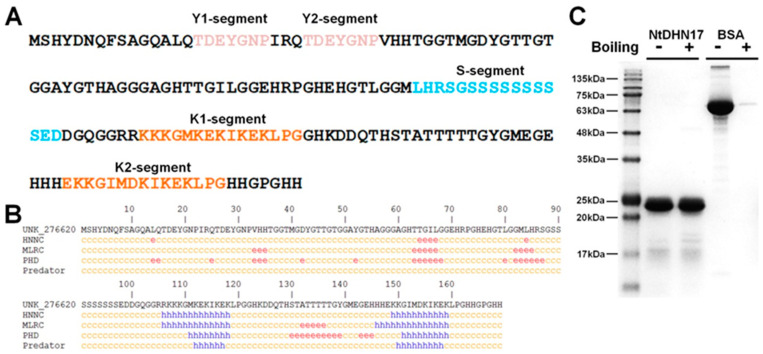
Schematic diagram of the deduced full-length NtDHN17 protein, the secondary structure analysis and heat-induced aggregation assay of NtDHN17. (**A**) NtDHN17 is an Y_2_SK_2_-type dehydrin; two typical K-segments were detected at the position 107~121 (K1) and 148~162 (K2); two Y-segments were at the position 15~22 (Y1) and 24~34 (Y2); one S-segment was at the position 89~101. (**B**) Different algorithms used to analyze the secondary structure of NtDHN17. The yellow letter “c” represents disordered region, the red letter “e” represents extended strand, and the blue letter “h” represents α-helix. (**C**) Heat-induced aggregation assay. The recombinant NtDHN17 and BSA (as control) were (+) or were not (−) boiled at 100 °C for 30 min. The samples were sedimented with centrifugation, and the supernatants were analyzed by 15% SDS-PAGE.

NtDHN17 protein was considered to be rich in hydrophilic amino acids, such as Gly (21.3%), His (10.7%) and Lys (7.7%), similar to the protein composition of other members of the dehydrin protein family [21]. Two typical K-segments were detected at positions 107~121 (K1) and 148~162 (K2), two Y-segments at positions 16~22 (Y1) and 26~32 (Y2) and an S-segment at position 84~99 (Figure 1A). Therefore, NtDHN17 is a Y_2_SK_2_-type dehydrin. The amino acid composition, content and hydropathic scale of NtDHN17 are shown in Table S1. The secondary structure of NtDHN17 was predicted with online tools: HNNC, MLRC, PHD and Predator, all of which gave similar results. The K-segment region forms an α-helix while the remining 70% of the amino acid sequence is disordered (Figure 1B, Table S2).

The highly hydrophilic properties of dehydrin proteins led them to be considered as intrinsically disordered proteins [21]. Disordered prediction was performed with the online tool PrDOS (Figure S1), and verified via heat-induced aggregation assay. NtDHN17 showed low mobility during SDS-PAGE analysis. Its predicted molecular mass was 17.8 kDa but it ran at about 25 kDa (Figure 1C). This high apparent molecular mass results from the highly hydrophilic properties which characterize IDPs [18,22]. Compared with a typical globular protein such as BSA, NtDHN17 was heat stable and retained its solubility during boiling for 30 min, meanwhile BSA was aggregated by the heat treatment (Figure 1C).

### 2.2. NtDHN17 Enhanced the Viability of E. coli Cells under Different Stress Conditions

**Figure 2:**
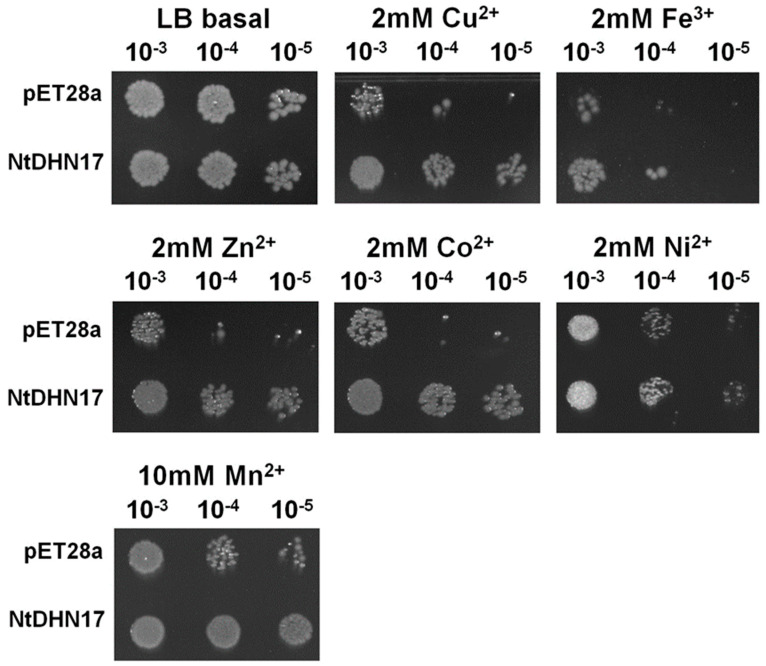
The growth performance of recombinant NtDHN17 on cell viability of *E. coli* transformant under metal stresses. Before spot assay, all cultures were adjusted to the same OD_600_. Each strain of the *E. coli* cells was diluted 10^−3^-, 10^−4^- and 10^−5^-fold and then were spotted on solid LB plates supplied with 2 mM CuSO_4_, 2 mM ZnCl_2_, 2 mM CoCl_2_, 2 mM NiSO_4_ or 10 mM MnSO_4_. Plates were cultured at 37 °C for 12 h.

To investigate the possible protective effects of recombinant NtDHN17, *E. coli* strains harboring *NtDhn17* or the empty control (BL/NtDHN17 and BL/pET28a) were treated with different stresses. Under the standard condition (LB medium without stress), growth of BL/NtDHN17 cells was similar to that of control BL/pET28a, indicating that the expression of NtDHN17 did not affect the growth of recombinant *E. coli* (Table 1). The viability of both recombinants decreased on culture with 2 mM CuSO_4_, 2 mM FeCl_3_, 2 mM ZnCl_2_, 2 mM CoCl_2_, 2 mM NiSO_4_ or 10 mM MnSO_4_ as a metal ion stress; 20% PEG600 and 500 mM mannitol as an osmotic stress; 500 mM NaCl and 500 mM KCl as a high salinity stress; or 4 mM H_2_O_2_ as an oxidative stress. However, BL/NtDHN17 cells showed better growth than BL/pET28 cells in the presence of exogenous metal ions, especially when divalent metal ions Cu^2+^, Zn^2+^ or Mn^2+^ were present (Table 1, Figure 2). The resistance to stress conferred by NtDHN17 in this study was Mn^2+^ ≈ Cu^2+^ ≈ Zn^2+^ ≈ PEG600 > Co^2+^ ≈ Ni^2+^ ≈ mannitol > NaCl ≈ KCl > Fe^3+^ ≈ H_2_O_2_.

Spot assays were used to ascertain cell viability of *E. coli* under metal ions stresses. Cell viability declined under copper treatment, but BL/NtDHN17 showed better growth than BL/pET28a at the same dilution ratio, suggesting that the over-expression of NtDHN17 reduced *E. coli* sensitivity to copper ion toxicity (Figure 2). Similar growth performances were seen with other metal stresses, Fe^3+^, Zn^2+^, Co^2+^, Ni^2+^ and Mn^2+^ (Figure 2).

### 2.3. Cellular Copper Content Increased in E. coli under Copper Toxicity

**Figure 3:**
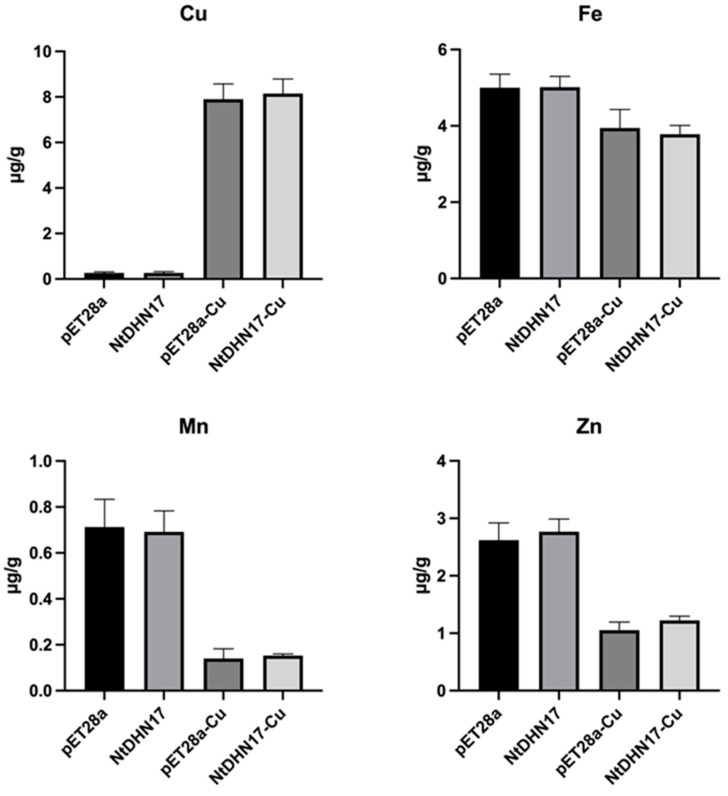
Cellular copper content increased in *E. coli* under copper toxicity. BL/pET28 and BL/NtDHN17 were grown in LB liquid medium with or without 5 mM CuSO_4_. Intracellular levels of copper, iron, manganese and zinc were determined by ICP-MS according to GB 5009.268-2016, issued by the National Standard Substances Center of China.

The effect of increased copper in the medium on the intracellular level of copper was determined by ICP-MS (Inductively Coupled Plasma Mass Spectrometry). BL/pET28 and BL/NtDHN17 were cultured with or without 2 mM CuSO_4_. *E. coli* cells stressed with copper accumulated remarkable levels of intracellular copper content (Figure 3), and we noticed that there was no difference between BL/pET28 and BL/NtDHN17 cells exposed to copper. We also measured the levels of iron, manganese and zinc, since altered intracellular copper level might affect the homeostasis of other metals. As shown in Figure 3, accumulated levels of copper in *E. coli* cells resulted in decreased levels of iron, manganese and zinc in both BL/pET28 and BL/NtDHN17 cells, and there was no significant difference between BL/pET28 and BL/NtDHN17 cells.

### 2.4. NtDHN17 Did Not Bind Cu^2+^ and Cu^2+^ Did Not Promote Self-Aggregation of NtDHN17

**Figure 4:**
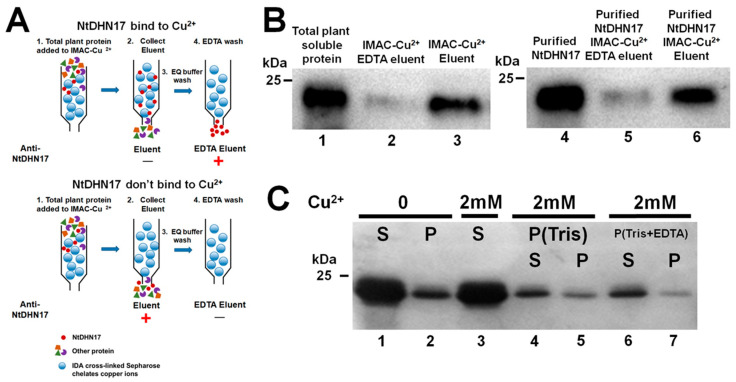
Cu^2+^-binding properties of the NtDHN17 protein. (**A**) Schematic diagram of two binding results between the immobilized metal ion affinity chromatography (IMAC)-Cu^2+^ with NtDHN17. (**B**) Using IMAC and western blotting to investigate Cu^2+^-binding property with native NtDHN17 and recombinant 6 × His-tag removed NtDHN17. Total soluble proteins from copper-treated tobacco leaves and recombinant NtDHN17 were applied to IMAC columns chelating with Cu^2+^ to bind for 1 h at room temperature, and then eluents were collected (Eluent). After being washed with the EQ buffer, an aliquot of EDTA was loaded to the columns to elute potential Cu^2+^-bound proteins (EDTA eluent). Thirty milliliters of first step eluent (Eluent) or EDTA eluent (EDTA eluent) were analyzed by the standard western blotting using anti-NtDHN17 antisera. (**C**) Precipitation analysis of the self-aggregation of recombinant NtDHN17 triggered by Cu^2+^ in vitro. S: supernatants after centrifugation; P: precipitates after centrifugation. P (Tris): precipitates re-solubilized by Tris buffer. P (Tris + EDTA): precipitates re-solubilized by Tris buffer plus EDTA.

The His-Xn-His motif, previously reported to be involved in copper binding, is present at the C-terminus of NtDHN17 (Figure 1A) [7,23], indicating that NtDHN17 might bind copper ions. Immobilized metal ion affinity chromatography was performed to test the binding affinity between NtDHN17 and Cu^2+^ ions. Two binding results may occur, as shown in Figure 4A. If NtDHN17 is detected in the EDTA eluent, it indicates that NtDHN17 can bind Cu^2+^, since it will be retained in the column immobilized with Cu^2+^ and requires EDTA for displacement. However, if NtDHN17 is detected in the first eluent (Eluent), it indicates that NtDHN17 cannot bind Cu^2+^ and it cannot be retained in the Cu^2^-column (Figure 4A).

Total soluble proteins from 4- to 6-week-old copper-treated tobacco leaves were applied to IMAC columns chelating with Cu^2+^ under high ionic strength (1 M NaCl) at pH 7.4. Western blotting analysis showed that NtDHN17 was detected in the Eluent, not the EDTA eluent. The result indicated that the native NtDHN17 did not bind directly to copper ions to be retained in the Cu^2^-column (Figure 4B, Lane 3). The experiment was repeated with recombinant NtDHN17 (6 × His-tag removed) to exclude the possibility that native NtDHN17 might be bound to Cu^2+^ within the plant cell. Recombinant NtDHN17 was also detected in the Eluent (Figure 4B, Lane 6), confirming that NtDHN17 did not bind to Cu^2+^ in vivo or in vitro.

Recombinant NtDHN17 did not undergo self-aggregation in the presence of excess copper ions in vitro (Figure 4C), remaining in the supernatant after incubation with Cu^2+^ for 60 min (Figure 4C, Lane 3). Resuspension of the pellet in Tris-buffer or Tris-EDTA buffer resulted in recombinant NtDHN17 remaining in the soluble supernatant (Figure 4C, Lane 4 and 6). Incubation with EDTA to chelate the Cu^2+^ had little effect on the release of NtDHN17 to the soluble fraction from the insoluble fraction (Figure 4C, Lane 5 and 7).

### 2.5. Copper Induced Significant Protein Aggregation and NtDHN17 Had an Anti-Aggregation Effect under Copper Stress

**Figure 5:**
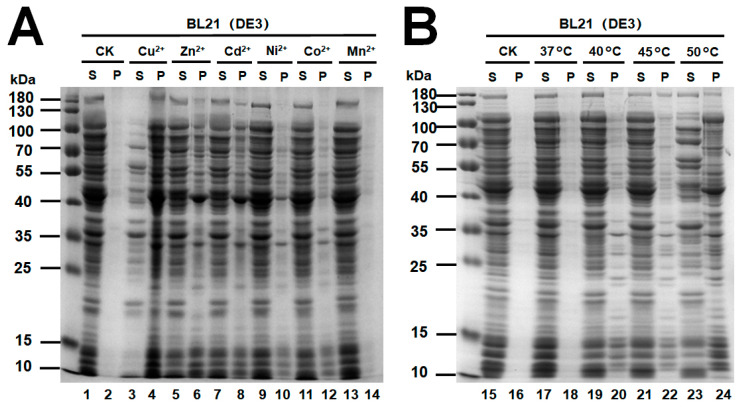
Stress-triggered protein aggregation in vitro. The soluble proteome of *E. coli* (30 μg each) was incubated with or without different stress factors: 5 mM Cu^2+^, 5 mM Zn^2+^, 5 mM Cd^2+^, 5 mM Ni^2+^, 5 mM Co^2+^, 10 mM Mn^2+^ for 60 min (**A**), and 45 °C and 50 °C for 15 min (**B**). Soluble (S) and aggregated proteins (P) from different treatments were further separated by centrifugation and analyzed by 12% SDS-PAGE.

The total soluble proteome was extracted from *E. coli* (BL21) and incubated with different divalent metal ions. SDS-PAGE analysis of the Cu^2+^-incubated soluble and insoluble fractions revealed a significant decrease in levels of soluble proteins (Figure 5A, Lane 4). Zn^2+^ and Cd^2+^ also caused accumulations of insoluble aggregated proteins (Figure 5A, Lane 6 and 8). The severity of the aggregation induced by distinct divalent metal ions tested in this study was Cu^2+^ > Zn^2+^ ≈ Cd^2+^ > Ni^2+^ > Co^2+^ > Mn^2+^ (Figure 5). The SDS-PAGE analysis of total soluble proteome after heat shock at 50 °C also revealed a significant decrease in the level of soluble proteins (Figure 5B, Lane 24).

**Figure 6:**
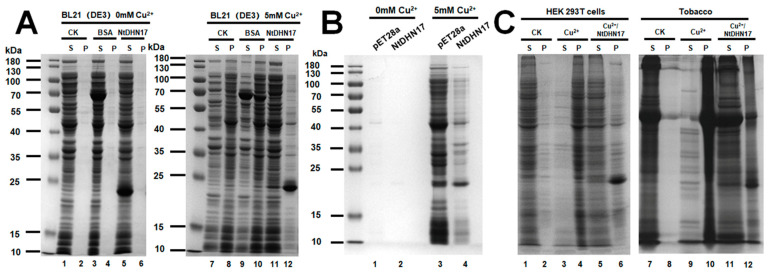
The recombinant NtDHN17 protein prevented protein aggregation induced by copper. (**A**) Thirty micrograms of soluble proteome from *E. coli* BL21 was incubated with 5 μg 6 × His-tag removed NtDHN17 at room temperature for 5 min. Then, 5 mM Cu^2+^ was added to *E. coli*. Soluble (S) and aggregated proteins (P) from different treatments were further separated at 15,000× *g* for 30 min at 4 °C, and analyzed by 12% SDS-PAGE. BSA was used as control. (**B**) Protein aggregates were isolated from BL/pET28 and BL/NtDHN17 cells treated with copper. The aggregated protein pellets from Cu^2+^-treated BL/pET28 and BL/NtDHN17 cells were suspended in 6M urea, and samples were analyzed by 12% SDS-PAGE and visualized by Coomassie blue staining. (**C**) Purified recombinant NtDHN17 can prevent soluble proteomes isolated from HEK 293 T cells and tobacco leaves from forming aggregates after copper treatment. Asterisk indicated recombinant NtDHN17 protein.

Copper toxicity resulted in protein aggregation and LEAs have been reported to prevent protein aggregation after heat, freezing or desiccation [24]. In the present study, the ability of NtDHN17 to prevent copper-induced protein aggregation was investigated in vivo and in vitro. *E. coli* soluble proteomes were first induced to aggregate by incubation with 5 mM CuSO_4_ for 60 min. Changes of the aggregated protein induced by copper treatment were analyzed by SDS-PAGE gels using globular protein BSA as control. Purified recombinant NtDHN17 and BSA did not cause protein aggregation under normal conditions (Figure 6A). However, in the presence of purified NtDHN17, the solubility of the *E. coli* proteome under copper toxicity was preserved (Figure 6A, Lane 11). The anti-aggregation protective property of recombinant NtDHN17 was verified in vivo with protein aggregates from BL/pET28 and BL/NtDHN17 cells. The SDS-PAGE analysis result of the aggregated proteins from BL/NtDHN17 cells that were treated with 5 mM CuSO_4_ revealed a significant difference when compared with BL/pET28 under same treatment (Figure 6B, Lane 3 and 4). Furthermore, the anti-aggregation effect showed by NtDHN17 was tested using total soluble proteomes extracted from tobacco and HEK 293 T cells. Soluble proteins from both plant and human cells aggregated after incubation with Cu^2+^ (Figure 6C, Lane 4 and 10). Purified recombinant NtDHN17 can prevent the formation of aggregates induced by copper treatment (Figure 6C, Lane 5 and 11). These observations demonstrated that NtDHN17 protein is able to function as a highly efficient anti-aggregation agent under copper toxicity, providing strong protection from the formation of protein aggregates.

### 2.6. Contributions of Different Segments to the Anti-Aggregation Effect

**Figure 7:**
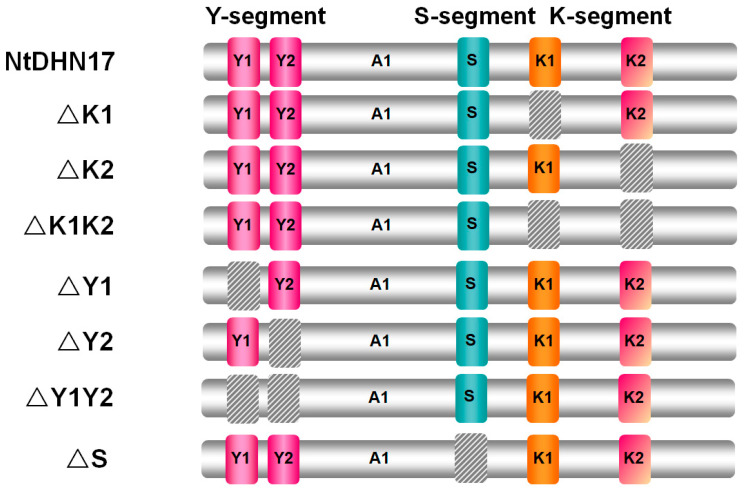
Schematic diagram of the truncate derivatives of NtDHN17. Seven different constructs were derived from NtDHN17 used in this work. Slash squares represent different conserved segment deletions. Positions of the conserved domain: the K-segments were at positions 107~121 (K1) and 148~162 (K2); the Y-segments were at positions 16~22 (Y1) and 26~32 (Y2); and the S-segment was at position 84~99.

The YSK-type is a major subgroup of dehydrins. To explore the contributions of those segments to the aggregation effect, **seven** truncated derivatives of NtDHN17 were constructed (Figure 7). The derivative polypeptides, ΔK1, ΔK2, ΔY1 and ΔY2, had either the first K-/Y-segment (K1 or Y1) or the second K-/Y-segment (K2 or Y2) deleted. ΔK1K2 and ΔY1Y2 lacked either two K- or Y-segments, and ΔS lacked the S-segment. DNA sequence analysis was used to confirm that, except for the segment(s) that had been removed intentionally (Figure 7), the remainder of NtDHN17 was intact in all the truncated derivative constructs.

The derivative polypeptides were analyzed with the online tool at https://web.expasy.org/protparam/, (accessed on 20 June 2021) (Table S3). The S-segment deletion mutants (ΔS) had similar features to full-length NtDHN17. Analysis of the isoelectric point, molecular weight and GRAVY showed that loss of the S fragment (ΔS) had little influence on the protein’s characteristics. K-segment deletion derivative polypeptides, ΔK1, ΔK2 and ΔK1K2, had molecular weights from 14.3 to 16.1 kDa, and the theoretical pI points were weakly acidic to neutral (6.0~6.50). The absence of the Y-segment in ΔY1, ΔY2 and ΔY1Y2 had a strong effect on the theoretical pI points which were neutral to weakly basic at 7.36~8.81. A 3D structural prediction performed by Phyre2 showed a random coil and two α-helices corresponding to the K1 and K2 segments, as expected, and the predicted 3D structure of ΔK1K2 showed several small loosely folded structures (Figure S2).

**Figure 8:**
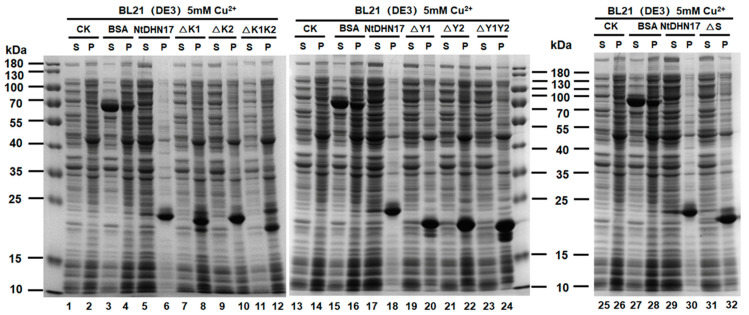
The contribution of different segments from NtDHN17 to protein aggregation induced by copper. The soluble proteome of *E. coli* (30 μg each) was incubated with 5 μg 6 × His-tag removed NtDHN17 and its truncated derivative polypeptides (ΔK1, ΔK2, ΔK1K2, ΔY1, ΔY2, ΔY1Y2, and ΔS) at room temperature for 5 min, then 5 mM Cu^2+^ was added to each sample and incubated for 60 min at room temperature. Soluble (S) and aggregated proteins (P) from different treatments were further separated at 15,000× *g* for 30 min at 4 °C, and analyzed by 12% SDS-PAGE. BSA was used as control. The protein bands were visualized by Coomassie blue staining.

Like purified recombinant NtDHN17, different truncated derivative polypeptides (6 × His-tag removed) did not cause protein aggregation under normal conditions (Figure S3). Different truncated derivative polypeptides showed different levels of anti-aggregation protection. Deletion of the first K1 segment (ΔK1) caused severe protein aggregation (Figure 8, Lane 8) which became worse when both K-segments (ΔK1K2) were deleted (Figure 8, Lane 11 and 12). Truncated derivatives lacking either Y segment (ΔY1 or ΔY2) or both (ΔY1Y2) showed a similar level of protection of the soluble proteome (Figure 8, Lane 20~24). Interestingly, the truncated polypeptide, ΔS, also showed a strong anti-aggregation effect (Figure 8, Lane 31 and 32), indicating that the S-segment might not be involved in the anti-aggregation effect. We also found, quite interestingly, that recombinant NtDHN17 and its truncated derivative polypeptides added to soluble proteome aggregated after incubation with copper, while globular protein BSA still remained in the supernatant (Figure 8 and Figure S3).

## 3. Discussion

Dehydrins are LEA group II proteins involved in responses to abiotic and biotic stresses [25]. The current study investigated functions of NtDHN17, the coding gene of which was isolated from dry mycelium of *Penicillium chrysogenum* induced tobacco leaf SSH cDNA library. Our major findings include the following. First, the heterologous expression of NtDHN17 enhanced the resistance of *E. coli* cells to copper toxicity. Second, IMAC analysis revealed that neither native NtDHN17 nor recombinant NtDHN17 bound to Cu^2+^. Third, NtDHN17 exhibited chaperone-like activity in suppressing the aggregation of soluble proteins induced by copper toxicity. Fourth, deletion of different conserved segments affected the chaperone activity of NtDHN17 to different extents. In conclusion, the protective role of NtDHN17 against copper toxicity in *E. coli* may be related to its anti-aggregation activity rather than to the scavenging of copper ions.

### 3.1. NtDHN17 Did Not Inhibit the Growth of E. coli and Enhanced the Viability of E.coli under Copper Toxicity

Sequence analysis indicated that NtDHN17 belongs to the Y_2_SK_2_ sub-group. Dehydrins are known to be rich in charged and polar amino acids, with high contents of Gly and His residues and lacking Cys and Trp residues [21]. NtDHN17 is rich in hydrophilic amino acids, thermostable and lacks a well-defined protein structure, all characteristics of IDPs [5]. Here in this study, the gene of interest is referred to as *NtDhn17* to emphasize its novel function relative to NtERD10B. The expression of NtERD10B was upregulated under chilling stress [26]. The expression of NtERD10B in *AcSnRK2.11* transgenic tobacco was upregulated under low-temperature conditions [27]. The expression of NtERD10B in PgDREB2A transgenic tobacco was also upregulated with different stress treatments [28]. To characterize the properties of the NtDHN17 protein, we used the heterologous expression in a prokaryotic system. It is helpful to overexpress LEA proteins heterologously in prokaryotic system to characterize their function [29,30,31]. Some dehydrins inhibit *E. coli* growth, including an *Arabidopsis thaliana* SK_3_-type dehydrin, ERD10 [32], a rice SK_3_-type dehydrin, RR46 [33] and a wheat YSK_2_-type dehydrin, DHN-5 [34]. Lysine-rich K-segments were found to be responsible for growth inhibition. The high proportion of positively charged Lys and Arg residues is reported to confer antimicrobial activity on proteins [35,36]. NtDHN17 is a neutral YSK-type dehydrin with K + R = 10.7% and did not inhibit *E. coli* growth under non-stress conditions. The recombinant NtDHN17 protein contributed better growth in *E. coli* cells under conditions of heavy metal toxicity and other stresses. Over-expression of *NtDhn17* in *E. coli* did not reduce the intracellular level of Cu^2+^, which indicated that *NtDhn17* enhanced the growth of *E.coli* not through a copper efflux system.

### 3.2. Neutral NtDHN17 Did Not Bind Cu^2+^ In Vivo or In Vitro

Some dehydrins, such as four Arabidopsis dehydrins, including AtHIRD11, can be isolated by Cu^2+^ and Ni^2+^ metal ion affinity chromatography [37,38] and AtHIRD11 may remove the Cu^2+^ inactivation of LDH to restore activity [14]. Metal binding properties are often associated with histidine-rich domains, such as double His (HH) sequences and His-X3-His motifs (HKGEH and HSGDH) [7,39]. NtDHN17 has both motifs at positions 34 (HH), 145 (HHH) and 163 (HHGPGHH). However, the results from immobilized metal affinity chromatography showed that neither native nor recombinant NtDHN17 bound Cu^2+^ in vivo or in vitro in the current study. MpDhn12 and AtHIRD11 bind Cu^2+^ and form self-aggregated after incubation with Cu^2+^, and could be released into the soluble fraction by EDTA treatment [8,39]. By contrast, copper did not induce NtDHN17 self-aggregation (Figure 3C). Previous reports have indicated that acidic dehydrins bound metal ions but neutral dehydrins, such as RAB18, did not [40]. NtDHN17 is a neutral dehydrin, thus we assumed that this feature may affect its metal-binding capacity and that binding to metal ions is not universal among YSK-type dehydrins, especially the neutral dehydrins.

### 3.3. NtDHN17 Showed Strong Protection to Copper Induced Protein Aggregation, an Effect Dependent on the K-Segment

High metal ion concentrations may promote aggregation and misfolding of soluble proteins [17,41]. Unregulated copper may target the central carbon metabolism in *E. coli*, and catalytic activities of GAPDH and IDH are known to be directly inhibited by copper [12]. Many proteins of glycolysis or the TCA cycle were stimulated to aggregate by 100 μM Cu^2+^ under aerobic conditions [10], as were peptides from ribosomal proteins and tRNA ligases, suggesting deceased protein synthesis as a consequence of copper toxicity [10,13]. The current study also demonstrated aggregations of soluble proteomes from *E. coli* cells, tobacco leaves and HEK 293 T cells under copper toxicity (Figure 5 and Figure 6C). Some LEA proteins are reported to function as molecular chaperones or molecular shields to prevent aggregation and/or restore improperly folded proteins under desiccation, heat shock or low temperature [42,43,44], and some dehydrins prevented denaturation of the *E. coli* soluble proteome in response to desiccation, heat shock or low temperature in vitro [24]. The present study showed that recombinant NtDHN17 suppressed copper-induced aggregation in vitro and in vivo (Figure 6). Quite interestingly, copper induced the aggregation of recombinant NtDHN17 in the presence of the *E. coli* soluble proteome (Figure 6A, Lane 12) but not in the absence of the proteome (Figure 6A, Lane 5 and 6), a result that was replicated with soluble proteomes from tobacco leaves and the HEK 293T cell line (Figure 6C). NtDHN17 is not self-aggregated by Cu^2+^, which might keep NtDHN17 function normal under copper stress, similar to interaction with other proteins. This is possible, because specific interactions between dehydrins and other proteins have been reported recently [45,46]. Interaction with partner proteins under stress conditions may induce conformational changes to NtDHN17 to be aggregated. Further research is needed to address how NtDHN17 functions in vivo.

The contribution of different conserved segments of NtDHN17 protein to the anti-aggregation function was investigated using the deletion mutation method. Seven truncated derivatives of NtDHN17 protein were produced by nucleotide sequence synthesis. The results showed that recombinant NtDHN17 suppressed copper-induced aggregation in vitro, an effect dependent on the K-segment but not on the Y or S segments. The ΔS derivative provided a strong protective effect similar to that of the NtDHN17, which indicated that the anti-aggregation effect was not associated with the phosphorylation of S-segment, because recombinant NtDHN17 is not phosphorylated in *E. coli* cells. Deletion analyses have previously shown dehydrin K-segment to maintain LDH or β-glucosidase activities under various stresses in vitro [15,47]. Our results suggested that, in concordance with other dehydrins, the general protective effect of NtDHN17 may be related to the amphipathic K segments.

In summary, the toxicity of excess copper ions to *E. coli* may be ameliorated by heterologous expression of the neutral YSK-subtype dehydrin, NtDHN17. NtDHN17 had a potent anti-aggregation effect during copper toxicity and may contribute to the stabilization of the cellular proteome. Further studies are necessary to address the protective mechanisms and chaperone role of NtDHN17 during copper toxicity and to identify proteins susceptible to copper-induced aggregation which may be binding partners of NtDHN17.

The data provided in our study suggested that functional heterogeneity is universal among the dehydrin family, and more research is needed to help us to see the whole picture about their roles in stress management.

## 4. Materials and Methods

### 4.1. Plant Material, Microbial Strains and Vectors

*Nicotiana tabacum* (L.) ‘K326′ was grown in a greenhouse (soil, 25 °C, natural light) with manual watering twice weekly and application of water-soluble fertilizer (N:P:K 20:20:20 + 0.5% *w/w* microelements) (Demei, Sichuan, China) once a month. Eight-week-old plants were treated with or without 7.5% DMP for 12 h, according to the method described by Zhong [48]. Young leaf tissues from DMP-treated plants and control plants were harvested or frozen in liquid nitrogen and stored at −80 °C until used. For copper treatment, four- to six-week-old tobacco plants were irrigated with 50 mM CuSO_4_ daily for three days.

*E. coli* DH5α and pMD19-T-simple (Takara, Dalian, China) were used for gene cloning and sequencing and *E. coli* BL21 (DE3) and pET28a (Invitrogen, Shanghai, China) for fusion protein expression.

### 4.2. Cloning the ORF of NtDhn17 and Bioinformatics Analysis of the NtDHN17 Protein

Total RNA was extracted from young tobacco leaves using RNeasy Plant Mini Kit (Takara, Dalian, China). First-strand cDNA was synthesized with 2 μg of total RNA using SuperScript II reverse transcriptase (Invitrogen, Shanghai, China). Primer pairs are listed in Table S4. All PCR products were cloned into the pMD19-T-simple vector and sequenced.

The amino acid composition, instability index and grand average of hydropathicity (GRAVY) of the deduced NtDHN17 and its truncated derivatives were predicted by an online tool ProtParam (https://web.expasy.org/protparam/, (accessed on 20 June 2021)). Protein hydropathy was analyzed with the Kyte–Doolittle Scale [49] and Hopp–Woods Scale [50]. Secondary structural analysis was carried out by HNNC, MLRC [51], PHD [52] and Predator [53]. Intrinsic disorder was predicted by an online tool, PrDOS [54]. The 3D structures of NtDHN17 and its truncated derivatives were predicted using an online tool, Phyre2 (http://www.sbg.bio.ic.ac.uk/phyre2/html/page.cgi?id=index, (accessed on 20 June 2021)).

### 4.3. Expression, Purification and Heat-Induced Aggregation Assay of Recombinant NtDHN17 Protein

Recombinant NtDHN17 was produced by the pET system and purified using the Ni Resin system (details in Supplementary Materials and Methods section). The 6 × His-tag was removed with a Thrombin Cleavage Kit (BioVision, Milpitas, CA, USA), following cleavage of NtDHN17 and thrombin removed by passing the reaction mix through a Heparin Sepharose column (BioVision, Milpitas, CA, USA). Protein concentration was determined by Bradford protein assay and verified by SDS-PAGE (15%-resolving/4%-stacking gels). Unless stated otherwise, the purified recombinant NtDHN17 used in this experiment was 6 × His-tag removed.

Resistance to heat-induced aggregation of NtDHN17 and BSA (10 μg each) was analyzed by boiling samples at 100 °C or keeping at room temperature for 30 min, followed by centrifugation at 20,000× *g* for 60 min at room temperature. Supernatants were analyzed by 15% SDS-PAGE. Unless stated otherwise, all results were derived from 3 independent replicates.

### 4.4. Protective Effect of NtDHN17 on E. coli under Different Stresses

Tolerance of *E. coli* BL21 (DE3) cells harboring a recombinant pET28a-NtDHN17 plasmid or the pET28a vector (empty control) to metal ions, osmotic stress, high salinity and oxidative stress conditions was evaluated using spectroscopic measurement and spot assay (details in Supplementary Materials and Methods). All results were derived from 3 independent replicates.

### 4.5. Analysis of Metals with ICP-MS

Each of 5 mL *E. coli* BL/pET28 and BL/NtDHN17 cells were grown in LB liquid medium at 37 °C for 12 h, diluted 50-fold with 80 mL fresh LB liquid medium and subcultured for about 2 h. Then, 1 mM IPTG was added, and the cells were subcultured at 37 °C until OD_600_ 0.5 was attained. Cells were treated with 5 mM CuSO_4_ for 30 min at 25 °C, and cells were then harvested by centrifuged at 4000 rpm for 10 min at 4 °C. The cell pellets were resuspended and washed with 10mL PBS buffer containing 50mM EDTA. The centrifugation and washing steps were repeated three times. The cell pellets were resuspended with 10 mL PBS buffer and harvested by centrifuged at 4000 rpm for 10 min at 4 °C. Cell pellets were digested with 2 mL Nitric acid at 100 °C for an hour. Concentration of copper, iron, manganese and zinc were analyzed using ICP-MS (NexION-2000B, PerkinElmer Waltham, MA, USA) according to GB 5009.268-2016, issued by National Standard Substances Center of China. All results were derived from 3 independent replicates.

### 4.6. Cu^2+^-Binding Properties and Self-Aggregation of NtDHN17

The interaction between Cu^2+^ and NtDHN17 in vivo was analyzed using immobilized metal ion affinity chromatography (IMAC) together with Western blotting. An IMAC-Cu^2+^ column (IMAC column charged with Cu^2+^) was prepared by applying 3 mL of 100 mM CuSO_4,_ washing of excess metal with 5 mL deionized water and column equilibration with EQ buffer (50 mM Tris-HCl, 1 M NaCl, pH 7.4). Total soluble proteins were extracted from copper-treated tobacco leaves at 4- to 6-weeks old by homogenizing in an ice-cold 25 mM Hepes–NaOH buffer (pH 7.5), containing 10 mM MgCl_2_, 0.7 M sucrose and 2 mM DTT with 1% cocktail protease inhibitor (Roche, NJ, USA) at a tissue: buffer ratio of 1:4 (*w/v*). Total soluble proteins (3 mL at 0.5 μg/μL) were loaded onto two IMAC-Cu^2+^ columns, incubated for 60 min at room temperature and eluents collected (eluent). Columns were washed with EQ buffer to elute unbound protein. A 5 mL volume of 10 mM EDTA was loaded onto the column to elute potential Cu^2+^-bound proteins (EDTA eluent). Aliquots of 30 μL eluent or EDTA eluent were analyzed by Western blotting using anti-NtDHN17 antisera produced by GenScript (Nanjing, China). The experiment was repeated with recombinant NtDHN17 (See Supplementary Materials and Methods for details).

The aggregation of recombinant Cu^2+-^bound NtDHN17 in vitro was detected by precipitation analysis, according to the method described by Mu [39]. Eight micrograms recombinant NtDHN17 were incubated in 10 mM Tris-buffer (pH 7.4) with or without additional 2 mM CuSO_4_ at 25 °C for 60 min and then centrifuged at 20,000× *g* at room temperature for 60 min. The supernatants (20 μL of the total 25 μL) and the sediments (5 μL of the total 25 μL) were analyzed by 15% SDS-PAGE to determine the relative soluble amount of NtDHN17 in each sample. For reversibility analysis of the precipitants, the sediments (5 μL) of NtDHN17 under 2 mM CuSO4 were diluted in 20 μL Tris-buffer or 20 μL Tris-buffer plus 10 mM EDTA and then centrifuged at 20,000× *g* at room temperature for 60 min. The supernatants (20 μL of the total 25 μL) and the sediments (5 μL of the total 25 μL) were analyzed by 15% SDS-PAGE to determine the relative amounts of resolubilized sediments of NtDHN17 in each sample. 

All results were derived from 3 independent replicates.

### 4.7. Preparation of E. coli Soluble Proteome and Stress-Triggered Aggregation of Proteins

*E. coli* BL21 (DE3) was grown in LB liquid medium at 37 °C for 12 h, diluted 50-fold with fresh LB liquid medium and subcultured for about 2.5 h until OD_600_ 0.6~0.8 was attained. Cells were harvested by centrifugation for 10 min at 10,000× *g* at 4 °C. Cell pellets were washed twice and resuspended in an ice-cold breakage buffer (1 × PBS buffer, pH 7.2~7.6) with 1% cocktail protease inhibitor (Roche, Branchburg, NJ, USA) and lysed by sonication at 4 °C. The lysate was centrifuged for 30 min at 15,000× *g* at 4 °C. The supernatant contains the soluble proteome and was quantified by the Bradford protein assay and diluted to 3.0 mg/mL.

Aliquots of 30 μg of soluble proteome were subjected to stress factors, as follows: 5 mM Cu^2+^, 5 mM Zn^2+^, 5 mM Cd^2+^, 5 mM Ni^2+^, 5 mM Co^2+^, 10 mM Mn^2+^ for 60 min or 45 °C or 50 °C for 15 min. Soluble (S) and aggregated proteins (P) were collected by centrifugation at 15,000× *g* for 30 min at 4 °C and analyzed by 12% SDS-PAGE. All results were derived from 3 independent replicates.

### 4.8. Preparation of Soluble Proteome from Tobacco and HEK 293T Cells

Total soluble proteins were extracted from 5-weeks-old untreated tobacco leaves by homogenizing in ice-cold 50 mM Hepes-NaOH buffer (pH 7.5), containing 150 mM NaCl, 5 mM EDTA, 0.5% Triton-100, 5% glycerol, 1 mM DTT, 1 mM PMSF and 1% cocktail protease inhibitor (Roche, Branchburg, NJ, USA) at a tissue: buffer ratio of 1:4 (*w*/*v*). The supernatant contains the soluble proteome and was quantified by the Bradford protein assay and diluted to 3 mg/mL.

Human embryonic kidney (HEK) 293T cells (American Type Culture Collection) were resuscitated from liquid nitrogen and cultured in Dulbecco’s Modified Eagle’s Medium (Solarbio Life Sciences, Beijing, China) with 10% fetal bovine serum in a 5% CO_2_ incubator. HEK 293T cells (1 × 10^5^ cells/mL) were collected and lysed in 100 μL ice-cold 50 mM Tris-HCl buffer (pH 7.5), containing 150 mM NaCl and 0.4% TritonX-100 at 4 °C for 30 min with occasionally gentle shakes. The lysate was centrifuged for 30 min at 15,000× *g* at 4 °C. The supernatant contains the soluble proteome and was quantified by the Bradford protein assay and diluted to 3 mg/mL. 

### 4.9. In vitro Protein Aggregation Assay

A 10 μL aliquot of total soluble proteome extracted from *E. coli* BL21 (DE3), untreated tobacco leaves and HEK 293 T cells (30 μg each) were incubated with 5 μg purified NtDHN17 (6 × His-tag removed) at room temperature for 5 min, respectively. Then, 5 mM Cu^2+^ was added to *E. coli* and HEK 293T cell extracts, and 10 mM Cu^2+^ was added to tobacco extracts, respectively. All the samples were incubated for 30 min at room temperature. S and P proteins were centrifuged at 15,000× *g* for 30 min at 4 °C, analyzed by 12% SDS-PAGE and visualized by Coomassie blue staining. All results were derived from 3 independent replicates.

### 4.10. Purification of Protein Aggregates Induced by Cu^2+^ from Whole E. coli Cells

The purification of protein aggregates induced by Cu^2+^ from whole cells was carried out according to the method described by Zuily [10]. Each of 5 mL *E. coli* BL/pET28 and BL/NtDHN17 cells were grown in LB liquid medium at 37 °C for 12 h, diluted 50-fold with 80 mL fresh LB liquid medium and subcultured for about 2 h. Then, 1 mM IPTG was added, and the cells were subcultured at 37 °C until OD_600_ 0.5 was attained. Cells were treated with 5 mM CuSO_4_ for 30 min at 25 °C, and cells were then harvested by centrifuged at 5000× *g* for 10 min at 4 °C. The cell pellets were resuspended and washed with 10 mL PBS buffer containing 50 mM EDTA. The centrifugation and washing steps were repeated three times. Each cell pellets were resuspended in 120 μL Buffer A [10 mM KH_2_PO_4_ (pH 6.5), 1 mM EDTA, 20% sucrose (*w*/*v*), 1 mg/mL lysozyme] and incubated for 30 min on ice. A total of 1080 μL of Buffer B [10 mM KH_2_PO_4_ (pH 6.5), 1 mM EDTA] was added to the samples, which were subsequently lysed by sonication on ice. After lysis, the samples were centrifuged at 5000× *g* for 15 min at 4°C to remove unbroken cells. Centrifugation at 15,000× *g* for 30 min at 4 °C was performed to isolate the insoluble cellular fraction, containing membrane and aggregated proteins. The pellets were resuspended in 1 mL of Buffer B by sonication and centrifuged at 15,000× *g* for 30 min at 4 °C. The pellets were resuspended by brief sonication in 960 μL of Buffer B, and 240 μL of membrane protein dissolution buffer (C500024, Sangon Biotech, Shanghai, China). After homogenization, centrifugation at 15,000× *g* for 30 min at 4 °C was performed to isolate the aggregated proteins. This washing, sonication and centrifugation steps were repeated twice to remove most of the membrane proteins. The pellets from Cu^2+^-treated BL/pET28 and BL/NtDHN17 cells were suspended in 60 μL of 6 M urea, and 10 μL samples were analyzed by 12% SDS-PAGE and visualized by Coomassie blue staining.

### 4.11. Construction of NtDHN17 Truncated Derivatives

Seven nucleotide sequences (Table S5) were synthesized at Sangon Biotech (Shanghai, China), digested with *Nhe* I and *Sac* I and inserted into the pET-28a vector to generate recombinant proteins lacking specific segments. Recombinant truncated polypeptides were expressed by a pET system and purified by a Ni Resin system. The 6 × His-tags were removed. Please see Materials and Methods section in Supplementary information for details.

In vitro protein aggregation assay was carried out using 5 μg purified 6 × His-tag removed polypeptides ΔK1, ΔK2, ΔK1K2, ΔY1, ΔY2, ΔY1Y2, and ΔS according to Section 4.9.

### 4.12. Statistical Analysis

Data are presented as mean ± standard error (SEM) for at least three independently replicated experiments. ANOVA and Duncan’s multiple range test were conducted using SPSS 16.0 software and a value of *p* < 0.05 considered to be statistically significant.

## Figures and Tables

**Table 1:** Growth rate of BL21/pET28a and BL21/NtDHN17 under different stresses.

Stress		OD_600_ (37 °C, 0 h)		OD_600_ (37 °C, 4 h)	
		BL21/pET28a	BL21/NtDHN17	BL21/pET28a	BL21/NtDHN17
No stress		0.105 ± 0.005	0.104 ± 0.005	1.008 ± 0.020 a	1.139 ± 0.145 a
Metal ions	2 mM CuSO_4_	0.105 ± 0.004	0.102 ± 0.003	0.526 ± 0.032 e	0.852 ± 0.044 bc
	2 mM FeCl_3_	0.104 ± 0.004	0.101 ± 0.002	0.355 ± 0.042 h	0.443 ± 0.048 f
	2 mM ZnCl_2_	0.102 ± 0.003	0.103 ± 0.003	0.482 ± 0.026 ef	0.833 ± 0.051 bc
	2 mM CoCl_2_	0.105 ± 0.001	0.103 ± 0.003	0.614 ± 0.024 cd	0.717 ± 0.012 d
	2 mM NiSO_4_	0.106 ± 0.004	0.104 ± 0.002	0.622 ± 0.011 c	0.767 ± 0.016 cd
	10 mM MnSO_4_	0.103 ± 0.003	0.103 ± 0.003	0.747 ± 0.035 b	0.924 ± 0.027 b
Osmotic stress	20% PEG600	0.106 ± 0.004	0.103 ± 0.003	0.727 ± 0.006 b	0.874 ± 0.010 b
	500 mM mannitol	0.103 ± 0.003	0.101 ± 0.001	0.574 ± 0.046 d	0.766 ± 0.054 cd
High salinity	500 mM NaCl	0.106 ± 0.003	0.103 ± 0.002	0.418 ± 0.006 g	0.568 ± 0.022 e
	500 mM KCl	0.106 ± 0.004	0.103 ± 0.003	0.440 ± 0.014 fg	0.544 ± 0.009 e
Oxidation	4 mM H_2_O_2_	0.104 ± 0.003	0.103 ± 0.005	0.277 ± 0.005 i	0.385 ± 0.008 f

Growth of 1 mM IPTG (Isopropyl-β-D-thiogalactoside)-induced *E. coli* cultures harboring NtDHN17 or control pET28a with a standard LB medium or in medium supplied with different stresses. The densities of different cultures were measured at 600-nm absorbance after 4 h culture. Error bars indicate standard deviation of the means from three independent measurements. Relationships among means were analyzed using a one-way ANOVA and Duncan’s multiple range test (*p* < 0.05). Means with same letter within a column (same treatment) were not significantly different.

## Data Availability

Not applicable.

## Supplementary Materials

The following supporting information can be downloaded at: https://www.mdpi.com/article/10.3390/ijms232315162/s1.
