# Supplementary material for: A YSK-Type Dehydrin from Nicotiana tabacum Enhanced Copper Tolerance in Escherichia coli
Source: Int J Mol Sci. 2022 Dec 2;23(23):15162. doi: 10.3390/ijms232315162 (PMC9737620; doi:10.3390/ijms232315162)
Supplement: Supplementary file 1 [file ijms-23-15162-s001.zip › New-A YSK dhn from Nt-Figures-SI-IJMS.pdf]

Supplementary Information – Figures

Figure S1

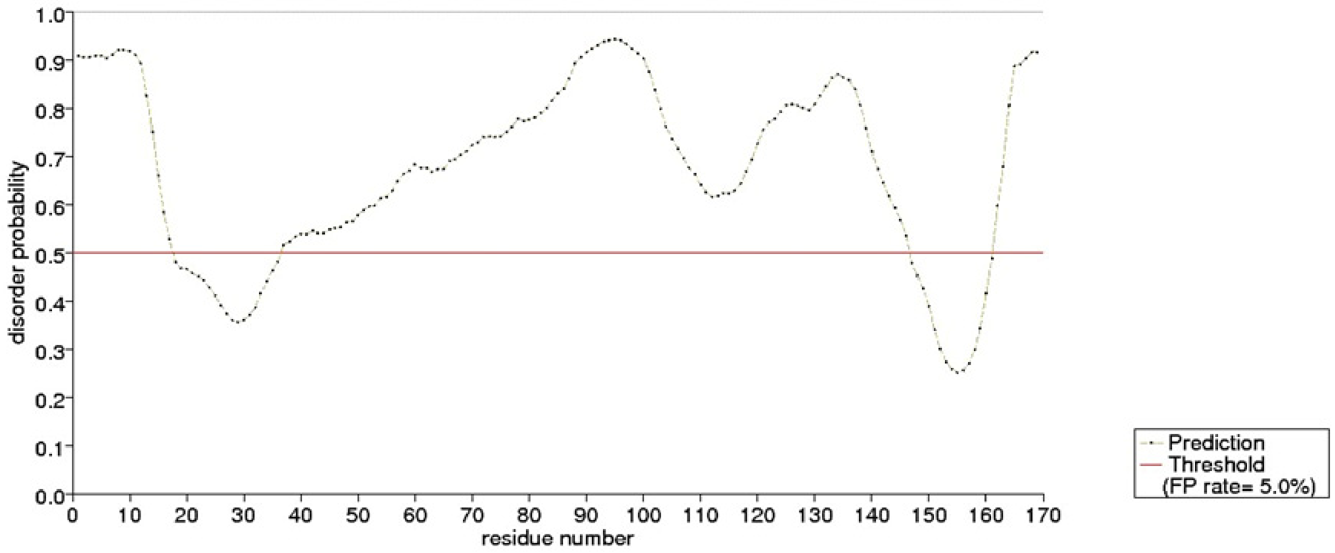

Figure S2

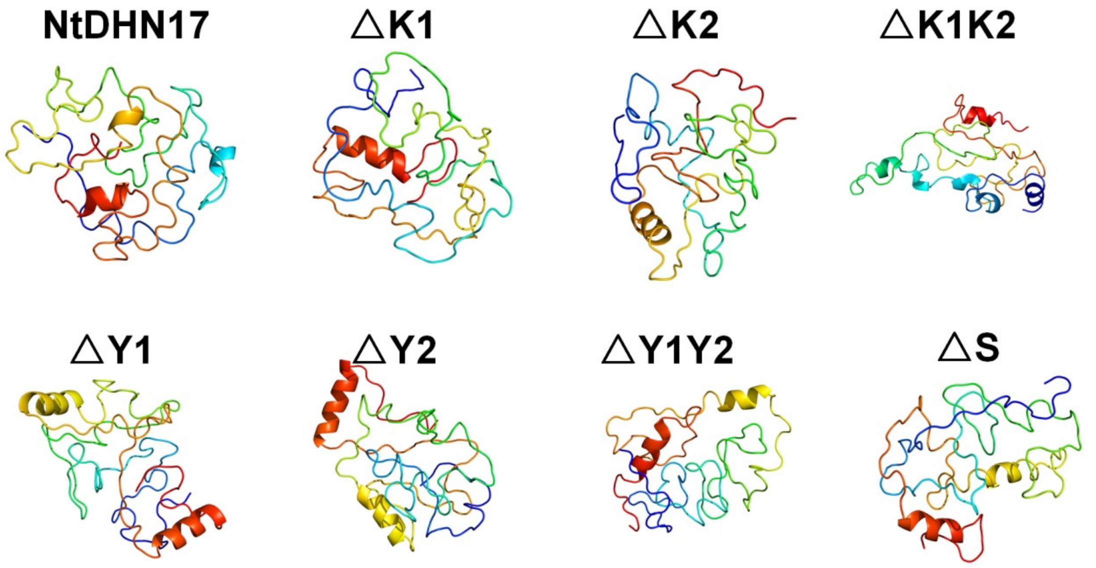

Figure S3

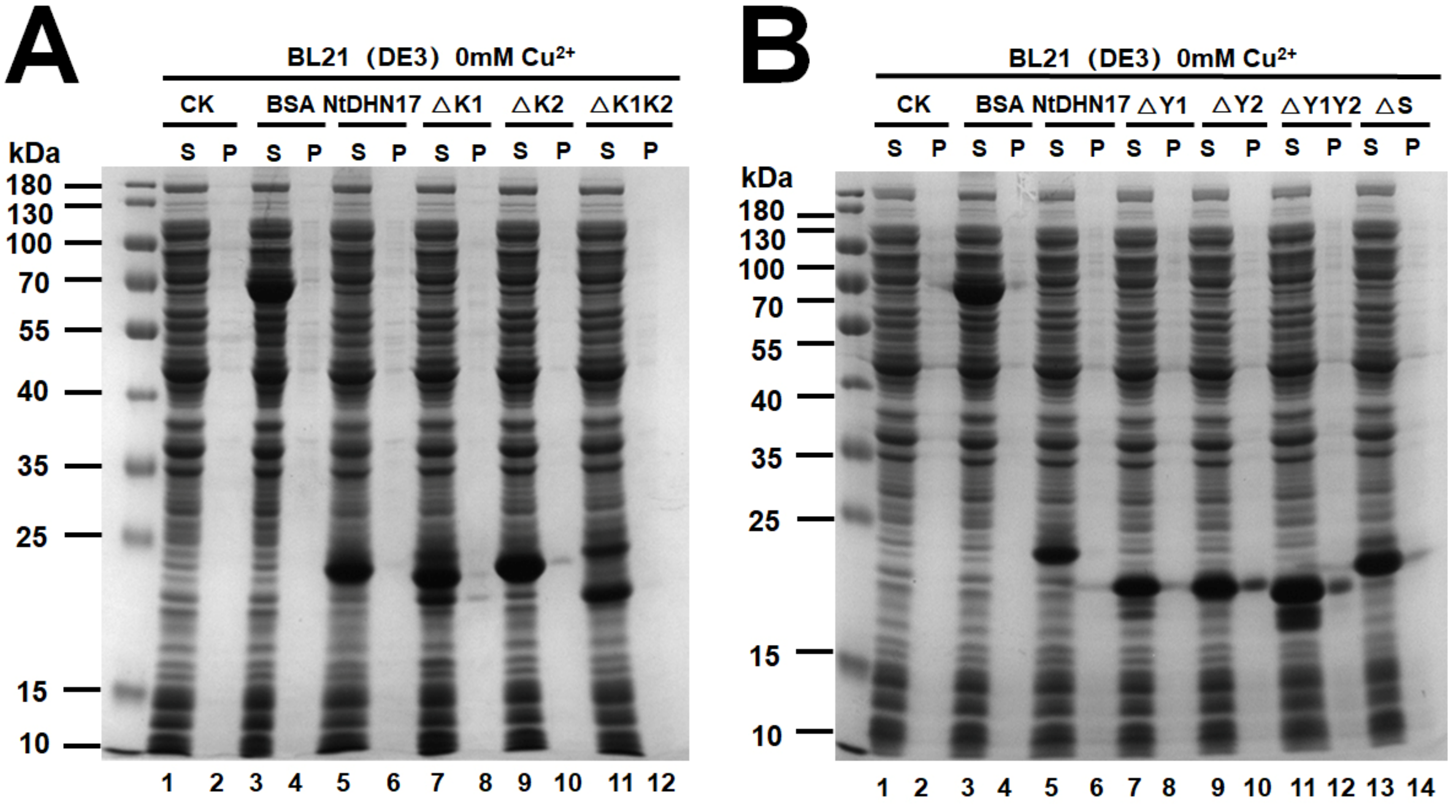
A
BL21 (DE3) 0mM Cu2+
CK BSA NtDHN17 △K1 △K2 △K1K2
S P S P S P S P S P S P
kDa
180
130
100
70
55
40
35
25
15
10
1 2 3 4 5 6 7 8 9 10 11 12
B
BL21 (DE3) 0mM Cu2+
CK BSA NtDHN17 △Y1 △Y2 △Y1Y2 △S
S P S P S P S P S P S P S P
kDa
180
130
100
70
55
40
35
25
15
10
1 2 3 4 5 6 7 8 9 10 11 12 13 14

Figure legends of Supplementary figures

Figure S1. The algorithm used to predict disorder with the deduced amino acid sequence of NtDHN17 protein was PrDOS. Disorder probability above 0.5 represented the disordered amino acid residues.

Figure S2. Prediction of three-dimension structures of NtDHN17 and its truncate derivatives.

Figure S3. The recombinants NtDHN17 protein and its truncate derivatives do not cause protein aggregation under normal condition. The soluble proteome of *E. coli* (30μg each) was incubated with 5μg 6×His-tag removed NtDHN17 and its truncated derivative polypeptides (△K1, △K2, △K1K2, △Y1, △Y2, △Y1Y2, and △S) at room temperature for 5 min. Soluble (S) and aggregated proteins (P) from different treatments were further separated at 15,000 *g* for 30 min at 4 °C, and analyzed by 12% SDS-PAGE. BSA was used as control. The protein bands were visualized by Coomassie blue staining. (A) △K1, △K2, △K1K2; (B) △Y1, △Y2, △Y1Y2, and △S.
