# Supplementary material for: A YSK-Type Dehydrin from Nicotiana tabacum Enhanced Copper Tolerance in Escherichia coli
Source: Int J Mol Sci. 2022 Dec 2;23(23):15162. doi: 10.3390/ijms232315162 (PMC9737620; doi:10.3390/ijms232315162)
Supplement: Supplementary file 1 [file ijms-23-15162-s001.zip › New-A YSK dhn from Nt-Tables-SI-IJMS.pdf]

Supplementary Information-Tables

Table S1. The amino acid composition, content and hydropathic scale of the NtDHN17 protein

Amino acid	Number	Content (%)	Kyte-Doolittle Scale	Hopp-Woods Scale
Glu	11	6.5	-3.5	3.0
His	18	10.7	-3.2	-0.5
Ala	6	3.6	1.8	-0.5
Lys	13	7.7	-3.9	3.0
Gly	63	21.3	-0.4	0.0
Tyr	6	3.6	-1.3	-2.3
Ser	13	7.7	-0.8	0.3
Asp	9	5.3	-3.5	3.0
Val	1	0.6	4.2	-1.5
Leu	6	3.6	3.8	-1.8
Phe	1	0.6	2.8	-2.5
Ile	5	3.0	4.5	-1.8
Thr	18	10.7	-0.7	-0.4
Met	6	3.6	1.9	-1.3
Pro	6	3.6	-1.6	0.0
Asn	3	1.8	-3.5	0.2

Table S2. Comparison of the content of secondary structure of NtDHN17 protein with different prediction algorithms.

Online algorithm	α-helix	Extended Strand	β-strand	Random coil
HNNC	14.2%	3.55%	0%	82.55%
MLRC	15.98%	10.06%	0%	73.96%
PHD	10.06%	20.12%	0%	69.82%
Predator	8.88%	0%	0%	91.12%

Table S3. Computational analyses of NtDHN17 and its truncated derivative polypeptides.

	Molecular weight （Da）	Number of amino acids	$No_{Arg+Lys}$ / (K+R)%	$No_{Asp+Glu}$	**G**ly (%)	Theoretical pI	GRAVY
NtDHN17	17773.14	169	18 / 10.7%	20	36 / 21.3%	6.81	-1.258
△K1	16048.96	154	11/ 7.1%	18	34 / 22.0%	6.19	-1.208
△K2	16077.06	154	13/ 8.4%	17	34 / 22.0%	6.50	-1.266
△K1K2	14352.88	139	6/ 4.3%	15	32 / 23.0%	6.00	-1.212
△Y1	16996.39	162	18 / 11.1%	18	35 / 21.6%	7.36	-1.223
△Y2	16996.39	162	18 / 11.1%	18	35 / 21.6%	7.36	-1.223
△Y1Y2	16219.63	155	18 / 11.6%	16	34 / 21.9%	8.81	-1.185
△S	16194.62	153	17/ 11.1%	18	35 / 22.9%	7.00	-1.263

The computational analysis of molecular weight, amino acid composition, theoretical pI and grand average of hydropathicity (GRAVY) of the full-length NtDHN17 protein and its truncated derivative polypeptides. 'Arg + Lys' represents the total number of positively charged residues and ' Asp + Glu ' represents the total number of negatively charged residues. GRAVY represents Grand average of hydropathicity.

Table S4. Primers used for cloning of *NtDhn17* ORF, construction of recombinants pET28a-NtDHN17 fusion protein expression vector

Name	Primers	Anneal Tm(°C)
NtDhn17 (for ORF)	F: 5′– AGTTCAGATTTTAATTAGTTTTGC -3′<br>R: 5′- TGCAACATTACAACGTACTTATT -3′	60
NtDHN17 (for protein)	F: 5′- CCTA*GCTAGC*ATGTCGCACTACGACAACCA -3′<br>R:5′- TTA*GAGCTC*CTAGTGGTGGCCAGGTCC -3′	60

F: forward primer, R: reverse primer

Nhe I and *Sac* I restriction sites (italic and underlined) were introduced to facilitate cloning in the corresponding sites of pET-28a vector to generate recombinant protein fused with an His-tag.

Table S5. Nucleotide sequences synthesized and deduced amino acid sequences (in bold letters) for construction of truncated derivatives fusion protein expression vector construction

Name of truncated derivative	Nucleotide sequences and **amino acid sequences**
△K1	CCTA*GCTAGC*ATGTCGCACTACGACAACCAATTTAGTGCAGGCCAGGCCTTGCAGACGGACGAATA<br>CGGCAATCCCATTCGTCAAACCGACGAATATGGGAACCCAGTCCATCACACTGGAGGTACCATGGG<br>AGACTATGGAACCACCGGAACAGGAGGTGCCTATGGAACTCACGCTGGCGGTGGCGCCGGCCACA<br>CCACTGGCATACTTGGTGGGGAACACCGTCCCGGCCATGAGCACGGTACTCTCGGTGGCATGCTCC<br>ACCGTTCTGGAAGCTCCAGCTCCAGCTCTAGCTCTTCGGAGGATGATGGACAAGGCGGAAGAAGG<br>GGTCACAAAGACGATCAGACTCATTCAACTGCAACAACTACGACTACCGGTTATGGTATGGAAGGG<br>GAGCATCATCATGAGAAGAAGGGAATCATGGACAAGATTAAGGAGAAGCTTCCTGGCCACCATGG<br>ACCTGGCCACCACTAG*GAGCTC*TAA<br><br>**MSHYDNQFSAGQALQTDEYGNPIRQTDEYGNPVHHTGGTMGDYGTTGTGGAYGTHAGGGAGH**<br>**TTGILGGEHRPGHEHGTLGGMLHRSGSSSSSSSSEDDGQGGRRGHKDDQTHSTATTTTTGYGME**<br>**GEHHHEKKGIMDKIKEKLPGHHGPGHH**
△K2	CCTA*GCTAGC*ATGTCGCACTACGACAACCAATTTAGTGCAGGCCAGGCCTTGCAGACGGACGAATA<br>CGGCAATCCCATTCGTCAAACCGACGAATATGGGAACCCAGTCCATCACACTGGAGGTACCATGGG<br>AGACTATGGAACCACCGGAACAGGAGGTGCCTATGGAACTCACGCTGGCGGTGGCGCCGGCCACA<br>CCACTGGCATACTTGGTGGGGAACACCGTCCCGGCCATGAGCACGGTACTCTCGGTGGCATGCTCC<br>ACCGTTCTGGAAGCTCCAGCTCCAGCTCTAGCTCTTCGGAGGATGATGGACAAGGCGGAAGAAGG<br>AAGAAGAAAGGGATGAAGGAGAAGATTAAGGAGAAATTGCCAGGAGGTCACAAAGACGATCAG<br>ACTCATTCAACTGCAACAACTACGACTACCGGTTATGGTATGGAAGGGGAGCATCATCATCACCATG

GACCTGGCCACCACTAG*GAGCTC*TAA

MSHYDNQFSAGQALQTDEYGNPIRQTDEYGNPVHHTGGTMGDYGTTGTGGAYGTHAGGGAGH TTGILGGEHRPGHEHGTLGGMLHRSGSSSSSSSSSEDDGQGGRRKKKGMKEKIKEKLPGGHKDDQ THSTATTTTTGYGMEGEHHHHHGPGHH

△K1K2

CCTA*GCTAGC*ATGTCGCACTACGACAACCAATTTAGTGCAGGCCAGGCCTTGCAGACGGACGAATA CGGCAATCCCATTCGTCAAACCGACGAATATGGGAACCCAGTCCATCACACTGGAGGTACCATGGG AGACTATGGAACCACCGGAACAGGAGGTGCCTATGGAACTCACGCTGGCGGTGGCGCCGGCCACA CCACTGGCATACTTGGTGGGGAACACCGTCCCGGCCATGAGCACGGTACTCTCGGTGGCATGCTCC ACCGTTCTGGAAGCTCCAGCTCCAGCTCTAGCTCTTCGGAGGATGATGGACAAGGCGGAAGAAGG GGTCACAAAGACGATCAGACTCATTCAACTGCAACAACTACGACTACCGGTTATGGTATGGAAGGG GAGCATCATCATCACCATGGACCTGGCCACCACTAG*GAGCTC*TAA

MSHYDNQFSAGQALQTDEYGNPIRQTDEYGNPVHHTGGTMGDYGTTGTGGAYGTHAGGGAGH TTGILGGEHRPGHEHGTLGGMLHRSGSSSSSSSSSEDDGQGGRRGHKDDQTHSTATTTTTGYGME GEHHHHHGPGHH

△Y1

CCTA*GCTAGC*ATGTCGCACTACGACAACCAATTTAGTGCAGGCCAGGCCTTGCAGATTCGTCAAACC GACGAATATGGGAACCCAGTCCATCACACTGGAGGTACCATGGGAGACTATGGAACCACCGGAAC AGGAGGTGCCTATGGAACTCACGCTGGCGGTGGCGCCGGCCACACCACTGGCATACTTGGTGGGG AACACCGTCCCGGCCATGAGCACGGTACTCTCGGTGGCATGCTCCACCGTTCTGGAAGCTCCAGCT CCAGCTCTAGCTCTTCGGAGGATGATGGACAAGGCGGAAGAAGGAAGAAGAAAGGGATGAAGGA GAAGATTAAGGAGAAATTGCCAGGAGGTCACAAAGACGATCAGACTCATTCAACTGCAACAACTA CGACTACCGGTTATGGTATGGAAGGGGAGCATCATCATGAGAAGAAGGGAATCATGGACAAGATTA AGGAGAAGCTTCCTGGCCACCATGGACCTGGCCACCACTAG*GAGCTC*TAA

MSHYDNQFSAGQALQIRQTDEYGNPVHHTGGTMGDYGTTGTGGAYGTHAGGGAGHTTGILGGE

HRPGHEHGTLGGMLHRSGSSSSSSSSSEDDGQGGRRKKKGMKEKIKEKLPGGHKDDQTHSTATTT
TTGYGMEGEHHHEKKGIMDKIKEKLPGHHGPGHH

△Y2

CCTA*<u>GCTAGC</u>*ATGTCGCACTACGACAACCAATTTAGTGCAGGCCAGGCCTTGCAGACGGACGAATA
CGGCAATCCCATTCGTCAAGTCCATCACACTGGAGGTACCATGGGAGACTATGGAACCACCGGAAC
AGGAGGTGCCTATGGAACTCACGCTGGCGGTGGCGCCGGCCACACCACTGGCATACTTGGTGGGG
AACACCGTCCCGGCCATGAGCACGGTACTCTCGGTGGCATGCTCCACCGTTCTGGAAGCTCCAGCT
CCAGCTCTAGCTCTTCGGAGGATGATGGACAAGGCGGAAGAAGGAAGAAGAAAGGGATGAAGGA
GAAGATTAAGGAGAAATTGCCAGGAGGTCACAAAGACGATCAGACTCATTCAACTGCAACAACTA
CGACTACCGGTTATGGTATGGAAGGGGAGCATCATCATGAGAAGAAGGGAATCATGGACAAGATTA
AGGAGAAGCTTCCTGGCCACCATGGACCTGGCCACCACTAG*<u>GAGCTC</u>*TAA

MSHYDNQFSAGQALQTDEYGNPIRQVHHTGGTMGDYGTTGTGGAYGTHAGGGAGHTTGILGGE
HRPGHEHGTLGGMLHRSGSSSSSSSSSEDDGQGGRRKKKGMKEKIKEKLPGGHKDDQTHSTATTT
TTGYGMEGEHHHEKKGIMDKIKEKLPGHHGPGHH

△Y1Y2

CCTA*<u>GCTAGC</u>*ATGTCGCACTACGACAACCAATTTAGTGCAGGCCAGGCCTTGCAGATTCGTCAAGTC
CATCACACTGGAGGTACCATGGGAGACTATGGAACCACCGGAACAGGAGGTGCCTATGGAACTCA
CGCTGGCGGTGGCGCCGGCCACACCACTGGCATACTTGGTGGGGAACACCGTCCCGGCCATGAGC
ACGGTACTCTCGGTGGCATGCTCCACCGTTCTGGAAGCTCCAGCTCCAGCTCTAGCTCTTCGGAGG
ATGATGGACAAGGCGGAAGAAGGAAGAAGAAAGGGATGAAGGAGAAGATTAAGGAGAAATTGC
CAGGAGGTCACAAAGACGATCAGACTCATTCAACTGCAACAACTACGACTACCGGTTATGGTATGG
AAGGGGAGCATCATCATGAGAAGAAGGGAATCATGGACAAGATTAAGGAGAAGCTTCCTGGCCAC
CATGGACCTGGCCACCACTAG*<u>GAGCTC</u>*TAA

MSHYDNQFSAGQALQIRQVHHTGGTMGDYGTTGTGGAYGTHAGGGAGHTTGILGGEHRPGHE
HGTLGGMLHRSGSSSSSSSSSEDDGQGGRRKKKGMKEKIKEKLPGGHKDDQTHSTATTTTGYG

	**MEGEHHHEKKGIMDKIKEKLPGHHGPGHH**
△S	CCTA*GCTAGC*ATGTCGCACTACGACAACCAATTTAGTGCAGGCCAGGCCTTGCAGACGGACGAATA<br>CGGCAATCCCATTCGTCAAACCGACGAATATGGGAACCCAGTCCATCACACTGGAGGTACCATGGG<br>AGACTATGGAACCACCGGAACAGGAGGTGCCTATGGAACTCACGCTGGCGGTGGCGCCGGCCACA<br>CCACTGGCATACTTGGTGGGGAACACCGTCCCGGCCATGAGCACGGTACTCTCGGTGGCATGGATG<br>GACAAGGCGGAAGAAGGAAGAAGAAAGGGATGAAGGAGAAGATTAAGGAGAAATTGCCAGGAG<br>GTCACAAAGACGATCAGACTCATTCAACTGCAACAACTACGACTACCGGTTATGGTATGGAAGGGG<br>AGCATCATCATGAGAAGAAGGGAATCATGGACAAGATTAAGGAGAAGCTTCCTGGCCACCATGGA<br>CCTGGCCACCACTAG*GAGCTC*TAA<br><br>**MSHYDNQFSAGQALQTDEYGNPIRQTDEYGNPVHHTGGTMGDYGTTGTGGAYGTHAGGGAGH**<br>**TTGILGGEHRPGHEHGTLGGMDGQGGRRKKKGMKEKIKEKLPGGHKDDQTHSTATTTTTGYGM**<br>**EGEHHHEKKGIMDKIKEKLPGHHGPGHH**

Nhe I and *Sac* I restriction sites (italic and underlined) were introduced to facilitate cloning in the corresponding sites of pET-28a vector to generate recombinant truncated derivative protein fused with an His-tag.
