# Supplementary material for: A YSK-Type Dehydrin from Nicotiana tabacum Enhanced Copper Tolerance in Escherichia coli
Source: Int J Mol Sci. 2022 Dec 2;23(23):15162. doi: 10.3390/ijms232315162 (PMC9737620; doi:10.3390/ijms232315162)
Supplement: Supplementary file 1 [file ijms-23-15162-s001.zip › New-YSK-type-SI-Materials and Methods-IJMS.docx.pdf]

Supplementary Information - Materials and Methods

Expression and purification of recombinant protein and truncated derivative polypeptides

A recombinant NtDHN17 protein and seven truncated derivative polypeptides were produced by pET system and purified using Ni Resin system. In brief, the open reading frame (ORF) of NtDHN17 was cloned, digested with *Nhe* I and *Sac* I and inserted into the pET-28a *Escherichia coli* expression system (Invitrogen, Shanghai, China). For recombinant proteins with or without the specified segments, seven nucleotide sequences (Table S5) were synthesized at Sangon Biotech (Shanghai, China). All sequences were digested with *Nhe* I and *Sac* I and inserted into the pET-28a vector to generate the truncated derivatives. Transformed *E. coli* BL21 (DE3) cells were grown in 2-YT liquid medium supplemented with 50 mg/L kanamycin and 34 mg/L chloromycetin at 37 °C for 12 hours. The bacterial cultures were diluted 100-fold using fresh 2-YT liquid medium and incubated until OD_{600} of 0.6. Protein expression, which was induced by the addition of 1 mM IPTG, proceeded for an additional 4 hours at 37 °C.

The recombinant NtDHN17 protein and truncated polypeptides △K1, △K2, △K1K2, △Y1, △Y2, △Y1Y2, and △S, were expressed as soluble polypeptides and affinity purified on Ni Resin FF (GenScript, Nanjing, China) under native conditions. The 6×His-tag was removed by Thrombin Cleavage Kit (BioVision, USA), following cleavage of NtDHN17 (or truncated polypeptides) and thrombin removed by passing the reaction mix through a Heparin Sepharose column (BioVision, USA). Recombinant 6×his-tag removal NtDHN17 protein and above truncated polypeptides were then concentrated and desalted by ultrafiltration with Ultrafree-MC filter units (3,000 NMWL, Millipore Corp). The concentrated proteins were the final purified protein or truncated polypeptides, and freeze-dried to keep at -80°C prior to use. Protein concentrations were determined by the Bradford protein assay and verified by 15%-resolving/4%-stacking 15% SDS-PAGE.

Protective effect of NtDHN17 protein on *E. coli* under different stresses

The *E. coli* BL21 (DE3) cells harboring a recombinant pET28a-NtDHN17 plasmid and the pET28a vector (empty control) were grown in liquid LB medium supplemented with 50 mg/L kanamycin and 34 mg/L chloromycetin at 37 °C for 12 hours. Subsequently, 1 mL cell culture

was inoculated into 100 mL of fresh liquid LB medium with 50 mg/L kanamycin and 34 mg/L chloromycetin at 37 °C for 2 hours. Then, 1 mM IPTG was added, and the cells were incubated at 37 °C until an OD_{600} of 0.4. The initial concentrations of subcultures were adjusted to the same OD_{600} (0.1) for each stress. To investigate growth kinetics of transformed *E. coli* strains under metal ions, osmotic stress, high salinity, and oxidative stress conditions, 10 mL of pre-IPTG-induced cultures was added to 40 mL stress-culture containing 1 mM IPTG and antibiotics. The stresses were induced by adding 2 mM $CuSO_4$, 2 mM $FeCl_3$, 2 mM $ZnCl_2$, 2 mM $CoCl_2$, 2 mM $NiSO_4$, or 10 mM $MnSO_4$ for metal ions; 20% PEG600 and 500 mM mannitol for osmotic stress; 500 mM NaCl and 500 mM KCl for high salinity; and 4 mM H_2O_2 for oxidative stress. The samples were cultured at 37 °C (220 rpm) for 4 hours, and OD_{600} was measured. Unless stated otherwise, all results were derived from 3 independent replicates.

Spot assay

E. coli BL21 (DE3) strain carrying recombinant NtDHN17 or the control vector, pET28a, were cultured in LB liquid medium with 50 mg/L kanamycin and 34 mg/L chloromycetin at 37 °C for 12 hours. Sub-culture was carried out by 50-fold dilution, adjustment to OD_{600} =0.2 and 1 mM IPTG was added before culture at 37 °C for 2 hours. Before spot assay, all cultures were adjusted to the same OD_{600} (0.2), and cultures were diluted to 10^{-3}-, 10^{-4}- and 10^{-5}-fold. A 6 μL volume of each samples were spotted onto separate LB basal plates containing 1 mM IPTG and metal ions, as follows: 2 mM $CuSO_4$, 2 mM $ZnCl_2$, 2 mM $CoCl_2$, 2 mM $NiSO_4$ or 10 mM $MnSO_4$. Plates were cultured at 37 °C for 12 hours. All results were derived from 3 independent replicates.

Cu^{2+}-binding properties and self-aggregation of NtDHN17

Cu^{2+}-binding of NtDHN17 *in vitro* was analyzed using IMAC together with Western blotting. One hundred micrograms of recombinant NtDHN17 (6×His-tag removed) were loaded onto two IMAC-Cu^{2+} columns, incubated for 60 min at room temperature and eluents collected (Eluent). Columns were washed with EQ buffer to elute unbound protein. A 5 mL volume of 10 mM EDTA was loaded onto the column to elute potential Cu^{2+}-bound proteins (EDTA eluent). Aliquots of 30 μL eluent or EDTA eluent were analyzed by Western blotting using anti-

NtDHN17 antisera produced by GenScript (GenScript, Nanjing, China).
